# Normal and tangential forces combine to convey contact pressure during dynamic tactile stimulation

**DOI:** 10.1038/s41598-022-12010-0

**Published:** 2022-05-17

**Authors:** David Gueorguiev, Julien Lambert, Jean-Louis Thonnard, Katherine J. Kuchenbecker

**Affiliations:** 1 Haptic Intelligence Department, Max Planck Institute for Intelligent Systems, Heisenbergstraße 3, 70569 Stuttgart, Germany; 2 Institut Des Systèmes Intelligents Et de Robotique, Centre National de La Recherche Scientifique (CNRS), Pyramide - T55/65, CC 173 - Place Jussieu 4, 75005 Paris, France; 3 Institute of Neuroscience, Université Catholique de Louvain, Avenue Mounier 53 – B1-53-04, B - 1200 Woluwe-St-Lambert, 1200 Brussels, Belgium

**Keywords:** Sensory processing, Perception

## Abstract

Humans need to accurately process the contact forces that arise as they perform everyday haptic interactions such as sliding the fingers along a surface to feel for bumps, sticky regions, or other irregularities. Several different mechanisms are possible for how the forces on the skin could be represented and integrated in such interactions. In this study, we used a force-controlled robotic platform and simultaneous ultrasonic modulation of the finger-surface friction to independently manipulate the normal and tangential forces during passive haptic stimulation by a flat surface. To assess whether the contact pressure on their finger had briefly increased or decreased during individual trials in this broad stimulus set, participants did not rely solely on either the normal force or the tangential force. Instead, they integrated tactile cues induced by both components. Support-vector-machine analysis classified physical trial data with up to 75% accuracy and suggested a linear perceptual mechanism. In addition, the change in the amplitude of the force vector predicted participants’ responses better than the change of the coefficient of dynamic friction, suggesting that intensive tactile cues are meaningful in this task. These results provide novel insights about how normal and tangential forces shape the perception of tactile contact.

## Introduction

Touching surfaces, grasping objects and manipulating tools are usual parts of daily life for most humans. During these interactions, we can effortlessly extract information about shape, material, and texture from almost every contact we make. When actively seeking particular haptic information, we are expert at using exploratory procedures to perform appropriate movements and maximize our perceptual performance ^1^. These movements enable us to gather the essential tactile cues required for fine perception of everyday objects ^2^, textures ^3^, or dexterous manipulation ^4^. The importance of tactile feedback is further highlighted by how challenging most daily tasks become when the sense of touch is lost ^5^ or when local anesthesia is applied ^6^.

Exploratory procedures necessarily generate forces and deformations on the skin; prior experiments have shown that these phenomena are essential sensory cues when active movements are performed ^7,8^. All types of afferents respond to slip events ^9^ and to frictional changes during passive sliding ^10^. Deformations that develop across the *stratum corneum* during these events create strain fields that depend on the intensity and direction of the applied force ^11,12^. When they are sufficiently large, the strains ultimately induce responses from mechanoreceptors ^9,13^. Furthermore, it has been shown that responses from a small population of afferents can accurately classify the exerted normal force and the frictional properties of textures ^14^. The signals from the activated sub-modalities then converge to the somatosensory cortex, where they activate several populations of cortical neurons that encode important perceptual dimensions of the tactile sense ^15^. Humans are therefore able to discriminate changes in force magnitude of 7–10% when the reference force is above 0.5 N and of 15–27% when it is under 0.5 N ^16^, as well as slips of less than 5 mm ^17^. Still, little is known about the cognitive mechanisms underlying these processes and how the physical attributes of contact force contribute to the perception of three-dimensional (3D) features through stereognosis.

Research on tactile perception of contact force has predominantly focused on understanding the role of frictional cues, which are related to tangential force. Humans have been shown to be extremely sensitive to changes in the tangential force ^18^, including nanoscale cues induced by molecular differences between materials ^19,20^. Moreover, finger-surface friction is known to modulate perception of surface roughness ^21,22^ and also mediate affective sensations such as the pleasantness of touch ^23^. Additional evidence of a possible predominant role of tangential force is provided by physiological and computational studies. Microneurographic recordings have demonstrated that the direction of the contact force vector affects the activity of all types of mechanoreceptors, which additionally exhibit a larger spiking response to the tangential component of the force ^24^. It was also shown that the finger-surface friction can be predicted by the geometry of the surface ^25^, and modelling of the interaction suggests that shear force is more informative than normal force for the recognition of shape during active tactile exploration ^26^. However, these studies analyzed solely contact mechanics measurements to assess the possible sensory relevance of these tactile cues; further psychophysical experiments are needed to understand whether the sense of touch relies on similar classification mechanisms.

Although human sensitivity to induced changes in the normal direction has been less studied than that for tangential force, scientists have shown that humans perceive normal force magnitude accurately ^27,28^. In a study that used the same experimental setup as this one, brief normal force changes were consistently detected with a just noticeable difference (JND) of 19% under both low and high surface friction ^29^. In that task, both tangential and normal cues were available, but the JND for tangential force varied across the conditions while normal force perception followed Weber’s law. Humans also accurately modulate normal force to optimally extract tactile features during a haptic task ^30^. Moreover, research on the perception of the 3D force applied on the fingertip has shown that humans accurately detect the direction of the force vector and discriminate differences in its tangential direction as small as 7.1° ^31^. Finally, sensory inputs from both force components may reinforce or disrupt each other during cognitive processes, as is the case for force, torque, and stiffness ^32^ despite being separately encoded in tactile afferents’ signals ^14,33^.

Thus, the extent to which the tactile sense relies on the normal and tangential force components for stereognosis is still unclear. In that context, it is especially important to investigate the sensory relevance of 3D force changes since forces have been shown to induce cues that can dominate the direct perception of surface topography ^7^. It is likely that sensory cues from both the tangential and normal force components are available to the tactile sense: the present study aims at investigating how these two tactile cues are integrated by humans within their perception of contact pressure during passive dynamic touch. To that end, we evaluate how humans perceive simultaneous changes of the normal and tangential force vectors when a flat, smooth surface is stroked across the stationary index finger. Passive stimulation was needed to control the forces experienced by participants with high enough precision. This type of stimulation removes the proprioceptive feedback related to the action ^8,34^ and prevents some adjustments that humans perform in active touch ^35^. However, several studies that delivered tactile cues on a moving surface have observed similar sensory thresholds for active and passive exploration ^36,37^.

**Figure 1:**
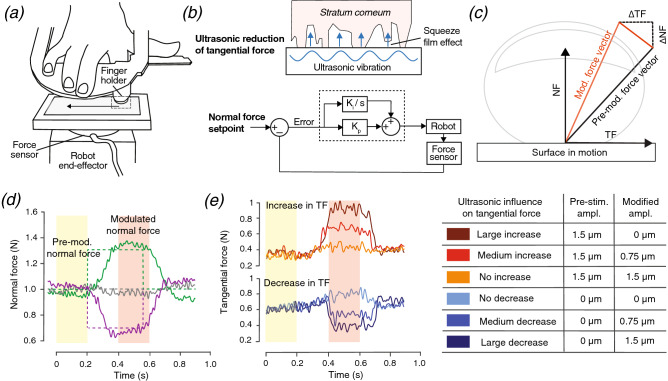
(**a**) The experimental apparatus used to independently modulate the normal force (NF) and tangential force (TF) that are applied on the index fingertip. (**b**) Upper: TF is modulated during sliding by vibrating the contact surface at an ultrasonic frequency (39 kHz) to create a microscale air film between the surface and the skin (squeeze film effect), hence reducing the finger-surface friction. Lower: diagram of the proportional-integral (PI) controller that enables the robotic platform to modulate NF according to a commanded pattern. (**c**) Illustration of the modulation of the contact-force vector when changes are induced compared to the pre-modulation NF and TF. TF is parallel to the contact surface, and NF is orthogonal to it. (**d**) Typical trials representative of the three normal force conditions: the briefly decreased normal force with ∆NF =  − 0.3 N (purple), the constant normal force of 1.0 N (gray), and the briefly increased normal force with ∆NF =  + 0.3 N (green). The setpoints are represented by the dashed lines. (**e**) Typical trials representing the six levels of change in TF. Three conditions start with no ultrasonic vibration, and the other three start with a constant 1.5 µm ultrasonic vibration. The changes in the intensity of the ultrasonic vibration and their impact on the finger-surface TF are displayed in the table on the right. One color is associated with each ultrasonic condition.

To achieve independent normal and tangential stimulations, we use custom-built equipment (Fig. 1a) that combines ultrasonic reduction of the elicited tangential force with a robotic platform capable of sliding a surface along a human fingertip while controlling the amplitude of the normal force (Fig. 1b). This apparatus enabled us to modulate the contact force vector (Fig. 1c) by generating different combinations of three amplitudes of normal force change (Fig. 1d) and six amplitudes of tangential force change (Fig. 1e). Importantly, the combinations of these conditions disrupted the naturally occurring correlation between the changes of the normal and tangential forces ^38^ to create stimuli that have not previously been studied. Due to the limited stimulation range of the ultrasonic apparatus, conditions with an increase of tangential force started from 1.5 µm ultrasonic lubrication, and those with a decrease started from natural finger-surface friction, as also done in ^10^. The recorded force vectors and answers enabled us to perform an objective analysis of the decision boundary that best fits our psychophysical results. In addition, we studied two metrics that are commonly used in haptics research, the coefficient of dynamic friction ^39,40^ and the amplitude of the 3D force vector ^41^. We tested these metrics because they could plausibly convey contact pressure through the perceived stickiness and the total pressure on the skin, respectively. The results of this study are fundamental for understanding the computational mechanisms of touch and fostering the further development of haptic devices that provide force feedback.

## Materials and methods

### Participants

Data were collected from 11 healthy volunteers aged between 27 and 53 (4 females). Ten participants self-reported their right hand as dominant, and one self-reported as ambidextrous. All participants performed the experiment with the index finger of their right hand. The ethics committee on human research of UCLouvain approved the study under reference 2019/03AVR/158. All participants gave written informed consent to David Gueorguiev, who conducted the human studies. The investigation conformed to the principles of the Declaration of Helsinki, and experiments were performed in accordance with relevant guidelines and regulations.

### Experimental setup

We used a custom robotic platform designed to apply controlled stimuli to the participant’s fingertip during passive dynamic touch. This platform is based on an industrial robot (four-axis SCARA Denso HS-4535G) that is able to translate in three orthogonal directions. Its position is servo-controlled with a position resolution of 15 µm by a factory controller at a frequency of 1 kHz, which enables exact control of the instantaneous speed of the sliding. The subject's index finger was fixed in a support that maintains a constant angle between the finger and the stimulating plate (Fig. 1a). A Mini40 load cell (ATI, USA) is mounted on the robot to measure the contact force vector; the force sensor’s single measurement resolution is 0.01 N in F_x_ and F_y_ (TF) and 0.02 N in F_z_ (NF). The normal force is controlled by a proportional-integral (PI) controller and was commanded to the baseline value of 1.0 N.

In addition to the control of the normal force by the robotic platform, the tangential force is modulated with an ultrasonic tactile display integrated with the acquisition and control chain of the robot. The display is based on a modified version of the STIMTAC ^42^. The full body of the stimulator is mounted to the force sensor of the robot, and the vibration amplitude of the device is controlled in closed loop. The implemented control ensures the stability of vibration amplitude with a resolution of 50 nm. The skin-plate interface was a polypropylene (PP) sheet, which was glued on the ultrasonically vibrating screen of the device. The baseline tangential force was either the natural finger-surface friction force or the friction force induced by a 1.5 µm ultrasonic vibration.

### Simultaneous modulation of tangential and normal force

For the three commanded force changes (− 0.3 N, 0 N, + 0.3 N), the robotic platform was able to achieve normal force changes of − 0.30 ± 0.02 N, − 0.01 ± 0.03 N, and 0.35 ± 0.03 N (mean ± SD), respectively. The changes exhibited a constant latency due to the inertia of the robotic platform but were reproducible and synchronized with the change in tangential force. In addition to the change in the applied normal force, the ultrasonic signal of the STIMTAC was commanded to change the tangential force in synchrony with the normal force modulation. Six conditions of ultrasonic stimulation were implemented. Three conditions started from a 1.5 µm ultrasonic vibration, which was then decreased during the modulation phase by 1.5 µm, 0.75 µm or 0 µm. Diminishing the vibration amplitude reduced ultrasonic lubrication and therefore increased the finger-surface friction. The other three conditions started without ultrasonic vibration, which then increased during the modulation phase by 1.5 µm, 0.75 µm or 0 µm, in order to decrease the finger-surface tangential force.

### Experimental procedure

The experiment combined the two techniques described above to achieve simultaneous and independent modulation of the two orthogonal components of the finger-surface contact force: the normal force and the tangential force. In a pre-modulation interval at the start of the trial, the robot slid the ultrasonic interface across the participant’s finger with a constant speed of 2 cm/s and a constant normal force of 1.0 N until 2.5 s after the start of the motion. This speed and force allowed for accurate control by the robotic platform and prevented artifacts such as stick–slip, which occur more often at higher speeds. This level of normal force is consistent with those observed in unrestrained roughness perception tasks ^43^. The exploration speed is at the low end of the range humans spontaneously use to explore a surface ^44^ and is consistent with values used in other studies ^10,18,43,45^. During the 0.5-s interval (modulated interval) following the initial 2.5 s of constant interaction, the normal contact force on the finger either decreased (− 0.3 N), increased (+ 0.3 N), or remained at the same value. After the brief change, the commanded normal force was set again to 1.0 N during the last interval of the platform’s motion.

The ultrasonic vibration could be moderately or greatly increased, moderately or greatly decreased or kept constant at the low or high starting value in synchrony with the change of the normal force. All combinations of the three normal force conditions and six ultrasonic conditions, which we selected to produce a large range of changes in NF and TF, were presented ten times in a randomized order for a total of 180 trials per participant, which took approximately 50 min. Participants started the experiment by performing six randomly chosen training trials to familiarize themselves with the stimuli. A one-minute break was provided in the middle of the experiment for stretching the hand. A few participants took one additional break of around 30 s when they felt tired in either the first or second half of the experiment.

After each trial, the participant had to report whether the contact pressure applied by the platform briefly increased or decreased around the middle of the trial. Answering that the pressure did not change was not allowed. The wording of the question relating to ‘contact pressure’ was chosen to avoid explicit reference to normal and tangential force while favoring the sensation related to the vertical action on the finger.

### Analysis of the force signals

The Mini40 six-axis force/torque sensor mounted between the robot and the ultrasonic plate was also used for the contact force measurements. The normal force component in z as well as the tangential force components in x and y were filtered by a low-pass (40 Hz) second-order Butterworth filter to remove the mechanical and electrical noise caused by the robotic platform; our sign convention for the study trials yields positive values for both NF and TF. We then used these filtered force signals to compute the relative changes in normal force and tangential force during the modulation period, compared to pre-modulation. We also calculate the amplitude *(A)* of the contact force vector,

$$A = \sqrt {F_{x}^{2} + F_{y}^{2} + F_{z}^{2} }$$

and the coefficient of dynamic friction *(µ)*, which is defined as the ratio between the finger-surface tangential force TF and the normal force NF,

$$\mu = \user2{ }\frac{{{\text{TF}}}}{{{\text{NF}}}} = \frac{{\sqrt {F_{x}^{2} + F_{y}^{2} } }}{{\left| {F_{z} } \right|}}$$

For each parameter, the pre-modulation measure was computed by averaging its values across the 200-ms interval prior to the start of the modulation, and the modulated measure was computed by averaging the values over the 200-ms interval in the middle of the modulated phase. The 150 ms during which the normal force is evolving toward its peak value and the 150 ms during which the normal force is returning to 1.0 N were excluded from the computation. For both metrics, we define the relative change as the ratio between the modulated measure and the pre-modulation measure.

### Statistical analysis

The decision to use parametric or non-parametric statistical methods on a given data sample was motivated by the Shapiro–Wilk normality test and the alignment of the Q-Q normality plot. The relevance of two-way analysis of variance (ANOVA) was probed by checking the normality of the unstandardized residuals from the independent variables. The statistical analyses of the study were performed with Graphpad Prism and IBM SPSS software. The support-vector-machine (SVM) analysis was performed with the Scikit-learn 1.0.2 Python library ^46^.

## Results

### Contact pressure perception

**Figure 2:**
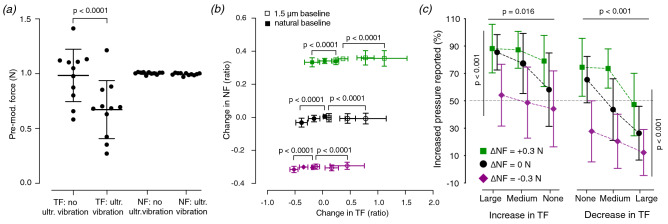
(**a**) The average tangential force (TF) and average normal force (NF) during the pre-modulation interval for all eleven participants in each condition, plus summary statistics (mean ± SD). (**b**) The change in TF and NF averaged across all participants for the 18 conditions of the experiment (**c**) The averaged answers across participants in the 18 conditions of the experiment (mean ± SD) Left: the conditions in which TF increased or stayed constant at a lower level during the modulated interval. Right: the conditions in which TF decreased or stayed constant at its natural higher level during the modulated interval.

Experiment participants were asked to report whether the contact pressure on their index finger had briefly increased or decreased during the interval in which the modulation of the force components occurred. Due to the limitations of the ultrasonic device, two pre-modulation conditions were used: one starting with natural finger-surface tangential force of 0.98 ± 0.25 N (mean ± SD) that was briefly decreased by ultrasonic lubrication, and one starting with maximum ultrasonic amplitude, hence a lower tangential force of 0.67 ± 0.27 N that was briefly increased during the modulated interval (see Fig. 2a). A statistical test confirmed that the difference between the TF of the two pre-modulation conditions was significant (paired t-test: *n* = 11, t = 14.21, df = 10, *p* < 0.0001). In contrast, the capacity of the robot to maintain a constant normal force of 1 N was not affected by the ultrasonic vibration (paired t-test: *n* = 11, t = 1.73, df = 10, *p* = 0.11).

Unlike NF, which was directly controlled by the robot with proportional and integral feedback, TF was indirectly modified by applying a pre-defined increase or decrease to the ultrasonic vibration amplitude; the effect of each change depends somewhat on the state of the skin, thereby producing slightly different frictional changes across trials. The variations of ultrasonic vibration were chosen to be large enough to produce different changes in TF. To verify that the levels of TF change were indeed statistically different from each other, we performed repeated measures ANOVA analyses with Greenhouse–Geisser correction on all six combinations (two baselines and three NF changes) that were modulated by three levels of ultrasonic vibration. We found all ANOVA analyses to be strongly significant with *p* < 0.0001 (Fig. 2b). In addition, post-hoc Tukey tests showed that the TF change was significant with *p* < 0.05 between all levels of ultrasonic modulation. These results show that participants experienced distinct TF change for each level of ultrasonic modulation.

Therefore, we further investigated how the perception of contact pressure is affected by simultaneous changes of normal force (NF) and tangential force (TF) by performing two two-way ANOVAs, which were separately implemented for the conditions in which TF decreased from its natural level and for those in which TF increased from a reduced level. The two ANOVA statistical tests were validated for the two types of conditions by a Q-Q plot of the unstandardized residuals as well as the Shapiro–Wilk normality test. The dependent variable in the analysis was the reported percentage of increased pressure, and two independent categorical variables were tested: the change of NF and the change of TF (See Fig. 2c). In the conditions starting with low friction, we found that both the change in NF (f(2) = 22.597, *p* < 0.001) and the change in TF (f(2) = 4.299, *p* = 0.016) had a statistically significant effect on the likelihood of the subject reporting a brief increase in contact pressure. In the condition starting with natural friction that briefly decreased, we also found a statistically significant effect on reporting a brief increase in contact pressure by both the change in NF (f(2) = 42.002, *p* < 0.001) and the change in TF (f(2) = 16.061, *p* < 0.001). The interaction between the two terms was not significant in either condition. Overall, the results show that a larger increase in TF or NF made participants more likely to report an increase of the contact pressure. Conversely, they more often reported a decrease of the contact pressure when TF or NF decreased during the modulated interval.

### Perception of simultaneous changes of the force components

**Figure 3:**
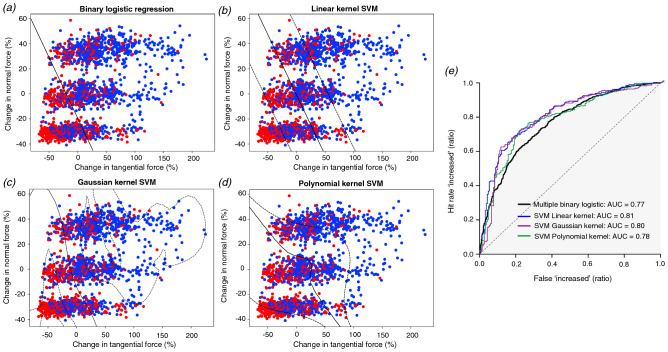
(**a**) The induced NF and TF changes during the modulated interval for all trials of the study and the classification line of the binary logistic regression. (**b**) Results for the linear kernel SVM with the addition of the 95% confidence interval boundaries. (**c**) Results for the Gaussian kernel SVM. (**d**) Results for the polynomial kernel SVM (degree = 3). (**e**) The receiver operator characteristic (ROC) curves obtained from the implemented classifiers.

Since the ANOVA statistical analysis showed significant effects of both TF and NF, we used linear and non-linear classification techniques to investigate the relative importance of the normal and tangential forces for human perception of contact pressure during dynamic tactile stimulation. In these analyses, the dependent variable was the perception of a decrease or increase, and the independent variables were the NF and TF changes generated in all trials of the study. At first, we performed a binary logistic regression because it is well suited for classification of binary answers and for modelling psychometric data. The analysis of the two independent variables with 20 iterations of the model showed a significant contribution of both variables (W = 55.7 and *p* < 0.001 for NF; W = 178.5 and *p* < 0.001 for TF). It achieved a 0.71 ratio of correctly classified answers (See Table 1 and Fig. 3a) with an area under the receiver operator characteristic (ROC) curve of 0.77 ± 0.01 (mean ± SD).

**Table 1:** Classification performance of the multiple logistic regression and the tested SVM classifiers on the NF and TF changes.

	Binary Logistic		Linear SVM		Gaussian SVM		Polynomial SVM	
	Precision	f1-score	Precision	f1-score	Precision	f1-score	Precision	f1-score
Decrease:	0.62	0.62	0.71	0.69	0.75	0.68	0.69	0.73
Increase:	0.77	0.76	0.75	0.76	0.73	0.78	0.80	0.75
W. average:	0.71	0.70	0.73	0.73	0.74	0.73	0.75	0.74

The precision is the ratio of correct classification, which was computed for each answer and as the weighted average. The f1-score is defined as the harmonic mean of precision and recall.

We explored further by performing an SVM analysis of the data with several kernels: linear, Gaussian, and third-degree polynomial. We implemented the classifications with a constant initial random state (42) to obtain repeatable results. 80% of the data were used to train the algorithm, and 20% were used as the test set. SVM slightly improved the classification rate compared to the binary logistic regression, especially the classification of the reported decreases. We also computed the boundary decision line and the 95% confidence interval for all kernels types (Fig. 3b–d); the boundaries for the Gaussian kernel approach suggest overfitting of the data. The areas under the curve also improved compared to the binary logistic regression (Fig. 3e). Overall, the third-degree polynomial fit performed best, and the contour line suggests that it improved classification by drawing a non-linear contour around the small changes to classify them as decreases. Here, the algorithm models a slightly higher reporting of decreases when changes in NF and TF are close to zero. In general, a relatively good classification performance could be achieved on the basis of NF and TF changes, and classification results mainly suggest linear perceptual mechanisms.

### The amplitude of the contact force and the coefficient of dynamic friction

**Figure 4:**
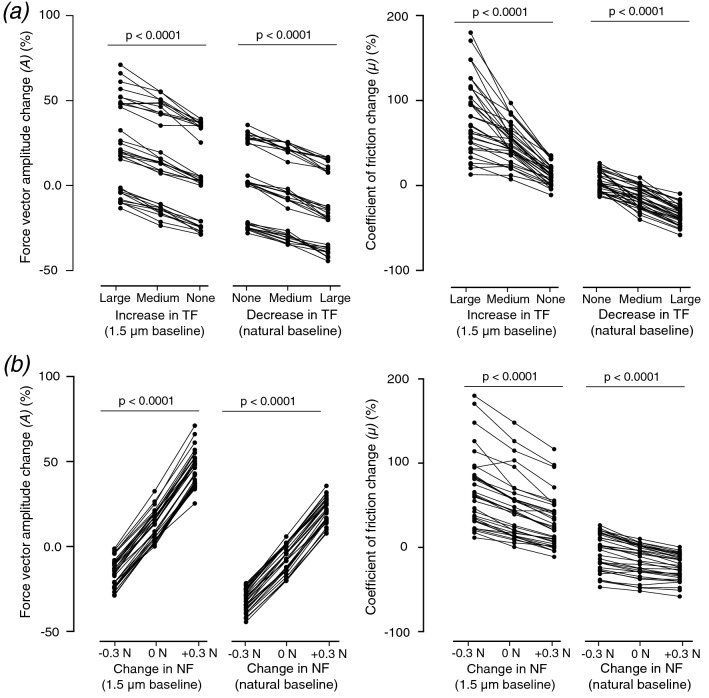
(**a**) Changes across TF conditions for the two metrics. Individual points represent the averaged change for one participant in a given condition. Lines connect conditions from the same participant with identical baseline and variation of normal force. (**b**) Same plot for the change across NF conditions. Lines connect conditions from the same participant with identical baseline and ultrasonic vibration.

In addition to the objective results from the classification algorithms, we surveyed two metrics that are often used in haptics studies and are plausible for this task: the amplitude of the 3D contact force *(A)* and the coefficient of dynamic friction *(µ)*, which is essentially inversely proportional to the angular change of the contact force vector. First, we tested whether these metrics showed significant variation when TF and NF changed. One-way repeated measures ANOVA with Greenhouse–Geisser correction on conditions in which TF was made to vary showed that changes in the metrics were significant between conditions with *p* < 0.0001 (Fig. 4a). The same statistical analysis was performed between the levels of NF change for the two metrics, and differences were also significant with *p* < 0.0001 for all comparisons (Fig. 4b). All post-hoc Tukey tests between conditions also showed *p* < 0.0001 significance. Overall, these results indicate that the changes induced by NF and TF are modifying these the metrics in a consistent manner.

**Figure 5:**
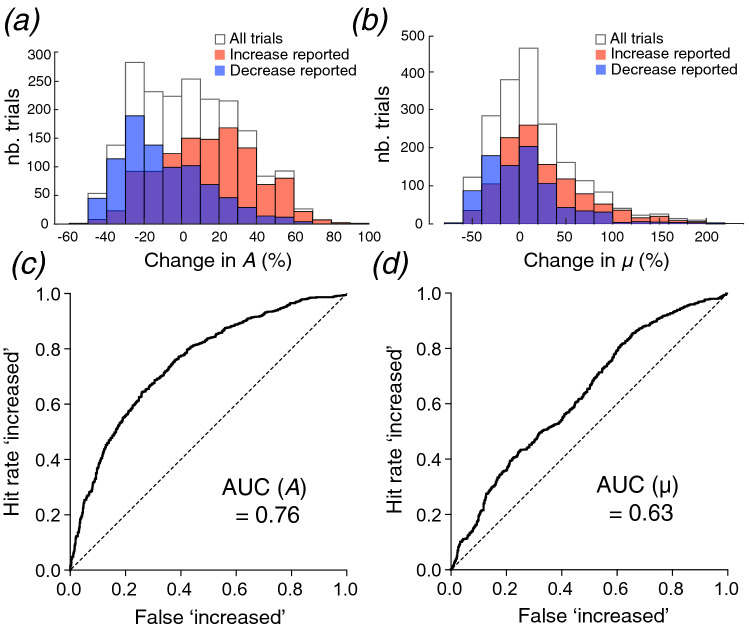
(**a**) Histogram of the number of answers with respect to the percentage change of the contact force vector amplitude *(A*). (**b**) Same histogram for the percentage change in the coefficient of dynamic friction *(µ)*. (**c**) Receiver operator characteristic (ROC) curve for the change in *A* across all participants. (**d**) Same ROC curve for the change in *µ*.

To test whether these metrics could be relevant to the perception of contact pressure, we performed binary logistic regressions in which participants’ answers were the dependent variable and the investigated metrics were probed as independent variables. For each metric, we computed the histogram of participants’ answers and the ROC curve across all trials of the study (Fig. 5). Histograms of the data from all trials show no excessive polarization, and their peaks are close to zero; thus, we did not observe significant distortions in the input spaces. For *A* (Fig. 5a), the histogram plot showed that the distribution of ‘increased’ answers mainly included positive changes in the amplitude of the force vector and the ‘decreased’ answer distribution mainly included negative ones. Moreover, the two distributions did not show a large overlap. On the other hand, the overlap between the reported increases and decreases was larger for the change in *µ* (Fig. 5b).

A binary logistic regression that computed ROC curves found an area under the curve of 0.76 ± 0.01 (mean ± SD) for the change in *A* (Fig. 5c) and 0.63 ± 0.01 for the change in *µ* (Fig. 5d). The average classification ratios (see Table 2) obtained with the one-dimensional binary logistic regression were respectively 0.70 and 0.63 for the changes in *A* and *µ*. Thus, the change of the coefficient of dynamic friction did not match well with participants’ answers, while performance with force vector amplitude is close to the classification achieved by objective analysis. These results suggest that intensive cues related to the amplitude change of the 3D force vector could have been used by participants to perform the task. Therefore, changes of the contact force amplitude probably elicit haptic cues that can contribute to the perception of contact pressure.

**Table 2:** Classification performance with a binary logistic regression for the changes in the contact force amplitude and the coefficient of dynamic friction.

	Contact force amplitude		Coefficient of dynamic friction	
	Precision	f1-score	Precision	f1-score
Decrease:	0.62	0.67	0.39	0.61
Increase:	0.76	0.72	0.81	0.63
Average:	0.70	0.70	0.63	0.62

## Discussion

The study results show that participants used both normal and tangential force components to assess changes in the contact pressure on the finger. Although the answer reported by a participant certainly depended on their particular interpretation of the term “contact pressure”, the feeling of contact pressure during dynamic stimulation seems to be conveyed not only by the normal indentation of the skin but also by its tangential stretch. A possible mechanical explanation of the important perceptual role of TF could be that, since the gross contact area is reduced when tangential force increases ^47,48^, the average pressure on the afferents in the contact area is increased. In addition, it seems that compressive strains induced by an increase in TF are larger than tensile ones ^12^, which could explain how an increase in TF would be perceived as a compression and hence an increase in contact pressure. A biomechanical explanation could also be that changes in tangential force induce rolling of the soft fingertip tissue, which then pushes toward the nail close to the normal direction. Moreover, our study considered the particular case of a surface that is perpendicular to gravity, but humans most often haptically explore objects like clothes, tools, and smartphones in complex orientations. Experience with such diverse facets of tactile contact might have shaped a more complex representation of contact pressure.

Thus, perception of the contact pressure during dynamic passive haptic stimulation is not mediated only by normal indentation or frictional cues but probably by the integration of all tactile cues induced by the force change. A unique aspect of this study was to use independent modulation of the tangential and normal force components to thoroughly investigate the mechanisms underlying the perceptual integration of the contact force exerted on the skin. The change in the coefficient of dynamic friction provided overlapping answering patterns and was subsequently a poor classifier of participants’ responses, which is surprising since it relates to the sensation of stickiness during dynamic touch ^49^. Participants probably mostly used intensive cues, which are not straightforwardly captured by computations relying on a ratio. Indeed, it is possible for the overall force to increase while the ratio between TF and NF decreases, and vice versa. In contrast, the contact force amplitude is an intensive cue that has rarely been examined in prior studies but was rather good at classifying participant answers. Overall, the best classification performance (75%) was achieved by the SVM with a polynomial kernel. It seems that the decision boundary is almost linear when changes are prominent but non-linear for very small changes. However, the limited classification improvement over SVM with a linear kernel (73%) makes it unclear whether this non-linearity stems from a sensory mechanism related to near-threshold intensities or from overfitting the dataset. Therefore, linear integration is the most plausible candidate for modeling the mechanisms underlying the perception of simultaneous changes in TF and NF.

Still, classification was not excellent, which is probably due to differences in the perceptual criteria between participants or even changes in decision-making strategy during the experiment. The comprehension of the task might also have had an influence on the perceptual strategy of participants. A task with a clear focus on either friction or normal force would likely have impacted the psychophysical results. The actual tactile mechanisms triggered by a force vector applied on the skin are unknown and might be either peripheral or central. On one hand, humans are quite accurate at perceiving the 3D contact force magnitude on their static fingerpad ^41^, and peripheral tactile afferents seem to encode the force vector ^14,24^. On the other hand, it has been observed that slowly adapting (SA) tactile afferents predominantly discharge when the skin is indented by an object ^50^. Humans can scale tangential force independently of the applied normal force ^51^, supporting the possibility of separate pathways in the peripheral nervous system. A possible candidate for this integrative process is the somatosensory cortex, in which other important tactile features such as speed and direction are encoded ^15^. Other alternatives also exist, such as integration by the cuneate nucleus, which has been shown to encode strain distributions ^52^.

A limitation of this study is the control of the patterns of ultrasonic vibration rather than the tangential force itself. Although the ultrasonic modulation was carefully controlled and identical across trials from the same condition, the actual change in TF slightly varied both across the trials of each participant and especially across participants, since the effect of ultrasonic vibration on TF depends on the physiology of the finger ^53^ and on phenomena such as stick–slip ^54^. Despite these variations, the differences between conditions were consistent and robust, which enabled new insights into the perception of independent NF and TF changes. Generating TF changes with exact values in force units could become possible with direct control of the finger-surface tangential force, for which promising preliminary results exist ^55^. Another limitation was the friction range of the ultrasonic device, which caused us to implement different baseline values of TF for the conditions in which TF increased and for those in which it decreased. Finally, our task featured stimuli that lasted 0.5 s in the middle of a passive stroke and required participants to consciously process the tactile cues. Since humans sense frictional cues within milliseconds of starting to press on a surface ^56^, the perceptual mechanisms mediating the results of our study might be different from the mechanisms underlying grasp. In future studies, we believe it will also be interesting to investigate a broad range of contact conditions and tactile tasks in order to confirm and quantify the perceptual mechanisms at work. Better knowledge of the interplay between tangential and normal force components will result in the definition of novel metrics and benefit the development of more realistic haptic feedback in tactile applications.

## Data Availability

All the datasets and scripts used within this study are accessible in the following Edmond repository: 10.17617/3.OVAO6R.
